# The Interplay of Parental Response to Anger, Adolescent Anger Regulation, and Externalizing and Internalizing Problems: A Longitudinal Study

**DOI:** 10.1007/s10802-021-00795-z

**Published:** 2021-03-12

**Authors:** Nantje Otterpohl, Elke Wild, Sophie S. Havighurst, Joachim Stiensmeier-Pelster, Christiane E. Kehoe

**Affiliations:** 1 Department of Psychology and Sports, Justus-Liebig-Universität Giessen, 35394 Giessen, Germany; 2 Department of Psychology and Sports, Bielefeld University, 33615 Bielefeld, Germany; 3 Department of Psychiatry, Mindful-Centre for Training and Research in Developmental Health, Melbourne, Australia

**Keywords:** Anger socialization, Anger regulation, Internalizing, Externalizing, Transdiagnostic, Longitudinal study

## Abstract

Numerous studies have reported substantive correlations between anger socialization, children’s anger regulation, and internalizing/externalizing problems. However, substantially less is known about the interplay among these constructs during the developmental stage of adolescence, and longitudinal studies on causal relations (i.e., parent-directed, adolescent-directed, or reciprocal effects) are rare. It is also unclear whether the development of internalizing and externalizing problems have similar causal relations. We collected three waves of longitudinal data (Grade 6, Grade 7, Grade 9) from multiple informants. A sample of *N* = 634 adolescents (mostly 11–12 years at Time 1; 50.6% male) and their parents (predominantly Caucasian with German nationality) completed questionnaires assessing parents’ responses to anger, adolescents’ anger regulation, and adolescents’ internalizing/externalizing problems at each wave. Comparisons of different cross-lagged models revealed reciprocal rather than unidirectional effects. However, we found more parent-directed effects with respect to the development of internalizing problems, whereas relations regarding externalizing problems were more adolescent-directed, i.e., adolescents’ externalizing problems and their anger regulation predicted changes in their parents’ responses to anger across time. Adolescent anger regulation was an important maintaining factor of parents’ responses to anger in later adolescence. Our findings suggest that assumptions regarding bidirectional relations should be emphasized much more in emotion socialization frameworks, particularly for the period of adolescence. Moreover, our study emphasizes the transdiagnostic importance of parents’ responses to anger for both externalizing and internalizing problems and also suggests different underlying mechanisms.

Emotional competence is central for healthy development in childhood and adolescence. Many skills in emotional competence are in place by middle childhood, however, research indicates that parents continue to play an important role in influencing an adolescent’s emotional understanding and regulation, which if poorly developed, are regarded as potential risk factors for both internalizing and externalizing problems (Eisenberg, 2020; Morris et al., 2007). One reason parenting continues to play an important role is that during this time adolescents experience negative emotions at greater intensity and show increases in mood variability (Bailen et al., 2019) providing ample opportunity for parental emotion socialization. However, it still remains an open research question what the interplay between emotion socialization, adolescents’ emotion regulation and adjustment looks like in adolescence. Because adolescents show heightened anger and as family conflicts increase, it is possible that this contributes to increases in unsupportive responses from parents, suggesting bidirectional processes may be involved. Closely related to this question, little is known about whether underlying mechanisms are similar for internalizing and externalizing problems.

In the present study, we seek to address these questions of directionality of relations between parents’ emotion socialization, adolescents’ emotion regulation and adjustment. By addressing these questions, we aim to generate greater knowledge about the importance of parental anger socialization as a key underlying risk/protective factor that may contribute to the improvement of the prevention and treatment of internalizing or externalizing problems.

## Emotion Socialization of Anger in Adolescence

As recommended by the functionalist approach (Gross & Thompson, 2007), we focused our study on one specific emotion instead of looking at emotions in general. Anger was selected because children and adolescents usually experience anger when their goal conflicts with external requests, thus blocking its attainment (Campos et al., 1994). Such situations are often accompanied by conflicts and aggressive behavior. Parents are likely to respond negatively to their adolescent’s anger, since they may have difficulty distinguishing between (always acceptable) emotions and (not always acceptable) behavior (Gottman & DeClaire, 1997) and thus view anger as negative and unpleasant.

In contrast, the functionalist approach views anger in a much more positive light: Anger is supposed to have an important signaling function (e.g., that a goal is not reached or one is treated unfairly) and provides feedback on the nature and quality of a relationship and the energy to change the current situation (Campos et al., 1994). As proposed in Gottman’s theory of emotion socialization (Gottman & DeClaire, 1997), supposedly negative emotions such as anger can offer a unique opportunity to create intimacy and closeness to the child. The discrepancy in the understanding of the desirability and functionality of anger makes the study of parental responses to this emotion particularly important.

Our study is embedded within the emotion socialization framework (Eisenberg et al., 1998; Morris et al., 2007) that understands the socialization of emotion as a process influencing a child’s learning regarding the experience, expression, and regulation of emotions. In addition to including the role of various child, parent, cultural, and contextual factors, this framework posits that parents’ emotion-related socialization behaviors (particularly their expression of emotions and reactions to children’s emotions) have a direct impact on children’s emotional arousal, emotion knowledge, and emotion regulation, which in turn, are expected to affect the child’s adaptation. Moreover, the framework acknowledges bidirectional relationships, including that a child’s level of emotional competence and mental health can reciprocally influence parents’ emotion-related socialization behaviors. For example, a child who is well-regulated in stressful interactions may elicit more supportive parental responses, whereas a child who shows greater arousal and less competencies in emotion regulation may elicit more unsupportive responses. In the past two decades, a large body of empirical evidence has been built supporting the role of emotion-related socialization behaviors (Eisenberg, 2020). To date, reciprocal processes proposed in the models have been scarcely examined in adolescence. Thus, our study aims to investigate reciprocal processes by investigating the emotion-related socialization behaviors of supportive vs. unsupportive parental responses to adolescents’ anger.

Parents supportive responses (e.g., accepting of emotions, encouraging of emotion expression, and validating of emotion experience) to adolescents’ anger may facilitate the expectation that adolescents will have their emotional needs met and may help to co-regulate the adolescent. Over time such responses are thought to facilitate the development of skills in anger regulation and mitigate adolescent externalizing and internalizing problems (Eisenberg et al., 1998; Gottman et al., 1996; Havighurst et al., 2015).

In contrast, unsupportive responses (such as dismissing, ignoring, minimizing or punishing emotions) may exacerbate adolescents’ emotional reactivity (Shenk & Fruzzetti, 2011), increase the likelihood of coercive interactions (as described in Patterson’s coercion theory, 2016, and related programs, e.g. parent management training or problem-solving skills training; Kazdin, 2003), leading to affective overarousal in adolescents (Eisenberg, 2020). Overarousal is often followed by difficulties in basic attentional processes (e.g., limited working memory capacities for focusing or shifting attention as needed) which are fundamental higher-order cognitive processes that assist with anger regulation (Eisenberg et al., 1998; Hoffman, 2000). Additionally, in emotionally charged situations, adolescents with poor emotional competence are likely to have biased perceptions and specific attributions that arouse anger and inappropriate reactions (e.g., aggression; Lemerise & Arsenio, 2000) which may manifest in externalizing problems (Dodge, 2006). In turn, these problems are likely to cause rejection by others and social withdrawal over time which may result in comorbid internalizing problems (Oh et al., 2020).

Unsupportive parental responses may also foster unhealthy beliefs about emotions that lead to the use of maladaptive emotion regulation strategies. For example, parents who ignore their teen’s anger may model avoidance of anger expression, and punishment of anger may communicate that emotions are unacceptable (Linehan, 1993) and should not be discussed or expressed, encouraging adolescents’ non-acceptance of emotions and use of anger suppression (Gottman & DeClaire, 1997). Such beliefs and strategies may contribute to the development of externalizing and internalizing problems.

In line with these assumptions, previous research has shown that when parents engage in supportive emotion socialization, adolescents exhibit better outcomes, including emotional competence, and fewer internalizing and externalizing problems, whereas inverse relations have been found for unsupportive emotion socialization (Eisenberg, 2020). A recent randomized control trial with parents of pre-adolescents found that reductions in unsupportive responses to their children’s anger, fear and sadness, resulted in decreased internalizing and externalizing problems (Havighurst et al., 2015; Kehoe et al., 2020). Moreover, Perry et al. (2020) demonstrated the predictive effects of parent supportive and unsupportive responses in early childhood (at 5 years of age) on pre-adolescent emotion regulation at age 10, which, in turn, predicted adolescent adjustment at age 15. However, although the study spanned an impressive longitudinal period, it only assessed each construct at one time point and thus did not focus on reciprocal relations.

## Reciprocal Relations Among Parents’ Anger Socialization, Anger Regulation, and Adjustment in Adolescence

Though scarcely studied, alternative assumptions on the directionality of relations between anger socialization, anger regulation, and adjustment are plausible for several reasons. The transition from early to mid-adolescence is characterized by many developmental transitions, such as the physical transitions at the beginning of puberty as well as social, educational, and motivational transitions associated with the transition from elementary to secondary school (Eccles & Wigfield, 1997). Adolescence is a key period in the maturation of the prefrontal cortex that underlies anger regulation processes; thus, adolescents might be particularly sensitive to anger socialization during this time (Bariola et al., 2011). There is evidence that adolescents experience a higher frequency and intensity of anger and also a maladaptive shift in the capacity to utilize adaptive emotion regulation strategies during early adolescence (Cracco et al., 2017). Moreover, adolescence is characterized by increased striving for independence from their parents and many day-to-day conflicts, which place parents at risk for dysregulation and decreased warmth (Steinberg, 2001). Parents have to rebalance their adolescents’ need for autonomy with emotional guidance, which, in turn, requires parents to recalibrate their responses to adolescents’ emotions. From the onset of puberty, parent–child interactions are likely to escalate more often, which is thought to increase the likelihood that parents will engage in reactive unsupportive responses (Maliken & Katz, 2013). Indeed, parents report higher levels of stress and negative affect during their child’s transition to adolescence (Steinberg, 2000). Thus, parent–child relationships tend to change profoundly in the adolescent years and it may well be that particularly externalizing problems, once manifested, also contribute to changes in parents’ anger responses, suggesting reciprocal or even adolescent-directed relations.

Only a few investigations have simultaneously examined the indicators of emotion socialization, emotion regulation, and adjustment in adolescents, and we found only three studies which examined reciprocal relations across time. All of these studies focused on the area of general parenting style rather than parents’ responses to anger. However, findings from these studies provide valuable indirect information about directionality of relations regarding other important indicators of parents’ emotion socialization (Morris et al., 2007; Eisenberg, 2020).

Using a three-wave cross-lagged panel study, Valiente et al. (2006) found parent-directed effects in the direction that the mothers’ expression of predominantly positive affect was positively associated with adolescents’ effortful control (a central component of successful anger regulation), which, in turn, was negatively associated with adolescents’ externalizing and internalizing problems. Interestingly, cross-lagged paths in the opposite direction were not significant. Similarly, Lengua (2006) found that parental rejection and inconsistent discipline were positively associated with externalizing and internalizing problems across three years from pre- to mid-adolescence (age 8–12 years old at Time 1). They assumed that adolescent temperament (fear, irritability, effortful control) would mediate these relations, but found that parenting and temperament predicted changes in each other, indicating additional reciprocal relations. Previous findings from our own study (Otterpohl & Wild, 2015) revealed reciprocal or adolescent-directed effects among parental responsiveness and psychological control, anger regulation, and internalizing/externalizing problems across two time points. In summary, there is currently some evidence that adolescent emotional and behavioral functioning may have an impact on parenting, however, these processes are yet to be investigated in relation to parents’ responses to anger.

## Transdiagnostic Implications of Parental Anger Socialization

Emotion regulation is understood to be an important underlying transdiagnostic mechanism in both internalizing and externalizing behavior problems (Beauchaine & Cicchetti, 2019). The term “transdiagnostic” means that the factor is theoretically proposed to be a shared mechanism explaining diverse problems. The identification of transdiagnostic risk or protective factors allows them to be targeted in interventions where benefits may be useful for different comorbid conditions because the underlying mechanisms have been the focus rather than the symptoms of the problem (Ehrenreich-May & Chu, 2013; Werner & Gross, 2010).

Previous research has shown that anger socialization and anger regulation are not only significantly associated with externalizing problems, but also with internalizing problems (e.g., Buckholdt et al., 2014; Folk et al., 2014). However, little is known about whether underlying directionalities of relations are similar or different for internalizing problems and externalizing problems. Providing support for the latter assumption, a two-wave cross-lagged panel study by Brenning et al. (2015) found a mixed pattern of results regarding the interplay of parents’ autonomy support and adolescents’ emotion regulation. Whilst clear parent-directed effects were found with respect to adolescents’ use of functional strategies and adolescents’ suppression of emotions, adolescent-directed effects were also found for insufficiently regulated emotions. This finding suggests that parent-directed effects may be more pronounced in adolescents with specific “inward” characteristics (e.g., overregulation of emotions, internalizing problems), whereas adolescent-directed effects may be more likely to appear for adolescents with “outward” characteristics (e.g. dysregulation of emotions, externalizing problems). Thus, it is possible that parent emotion socialization and adolescent emotion regulation may play a different (either determining, maintaining, or mediating) role for the development of internalizing and externalizing problems. However, to better understand their directionality of relations, a three-wave cross-lagged panel study would have been necessary. Moreover, as in the studies mentioned above, it is unclear whether findings on the role of autonomy support can be transferred to parental responses to emotions.

## The Present Research

The present study aimed to explore the directionality of relations between parental responses, adolescent anger regulation, and internalizing/externalizing problems during the transition from early to mid-adolescence. We conducted a three-wave longitudinal study with a large sample of German adolescents and their parents. Beyond concurrent relations and stability over time, we expected reciprocal effects of parents’ unsupportive anger socialization, adolescents’ dysfunctional anger regulation, and adolescents’ internalizing/externalizing problems. In addition, we expected a different interplay among these variables for internalizing and externalizing problems.

## Method

### Recruitment and Procedure

Data were drawn from a four-wave longitudinal project across four years (Grade 5 to Grade 9), supported by the German Federal Ministry for Education and Research. All data relevant for the present study (parent responses to anger, adolescent anger regulation, and internalizing/externalizing problems) were collected in the second (Grade 6), the third (Grade 7) and the fourth (Grade 9) measurement points, being Spring 2011, 2012, and 2014, respectively. No data were collected at Grade 8. Participants were recruited from two midsized towns in northern Germany, including neighboring villages. In Germany, students attend different tracks within the secondary school system that reflect academic aptitude. We first selected several schools representing the highest track (*Gymnasium*) and the lowest track (*Hauptschule*). All schools received a letter with information about the study and an invitation to participate. Altogether, 29 of 109 (26.6%) schools decided to participate (8 highest track and 21 lowest track schools). As the German highest track schools typically have a larger number of students than the lowest track schools, accordingly, the highest track students comprised a greater proportion of the overall participants.

Questionnaires were administered during class time to all adolescents who had provided informed assent and informed consent of their parents, which families had received together with an information letter before the survey and should return signed to the teacher. After filling out the questionnaire, adolescents received an envelope containing the parents’ questionnaire and were asked to take it home to their mother, or their father, who then completed the questionnaire at home. All families who completed both questionnaires, received a 15€ gift voucher. An ethics proposal was not submitted for the present study, since an institutional approval had already been obtained for a pilot study (April 2009; Bielefeld University) and the IRB decided that a new proposal submission was not necessary.

### Participants

Of the 1,763 families who were invited to participate in the study, 1,341 adolescents (76%) and 918 parents (52%) provided informed consent and filled out the questionnaires on the baseline measurement at Grade 6. Of these cases, the final sample was selected according to the following procedure: First, we selected all the families in which both adolescents and parents had participated at baseline measurement because we were interested in comparable multi-informant reports of adolescents’ internalizing and externalizing problems. In the next step, all adolescent-parent dyads who participated at all three waves were selected. To avoid extreme dropout, the cases in which either the adolescent or the parent questionnaire was missing at only one wave were also included. This procedure resulted in a final sample of *n* = 634 adolescent-parent dyads (69% of the 918 Grade 6 baseline dyads; see Table 1 for more specific information).

At baseline measurement, majority of adolescents (93.9%) were 11 or 12 years old (*Mage* = 11.77; *SD* = 0.56, range = 10–14 years). About half (50.6%) were male, 79.9% attended the highest school track, and 93.0% reported that German was always or often spoken in their family. For parents (fathers 8.7%; mothers 91.3%), 30.8% of all fathers, and 22.6% of all mothers, were in possession of a university degree; 50.0% of all fathers, and 63.4% of all mothers, were in possession of a training qualification, while 5.2% of fathers, and 6.3% of mothers, had no training qualification. Parents’ SES (Highest International Socio-economic Index of Occupational Status, *HISEI*; Ganzeboom et al., 1992) was slightly higher than the representative average in Germany in the year when the SES was assessed (*M* = 47.6; German Federal Ministry for Education and Research, 2008), with an average score of *M* = 55.62 (*SD* = 15.67) on a scale ranging from 16 (e.g., unskilled worker) to 90 (e.g., judge).

We used the German Strengths and Difficulties Questionnaire norms which are almost identical to the U.S. norms (Klasen et al., 2000) to describe the overall extent of psychological problems in the present sample. With respect to parent-reports of adolescents’ symptoms at Grade 6, 87.7% (Grade 7: 85.5%; Grade 9: 89.0%) of the adolescents were classified in the normal range, 6.2% (Grade 7: 7.5%; Grade 9: 5.8%) as borderline, and 6.1% (Grade 7: 7.0%; Grade 9: 5.2%) as clinically elevated. With regard to adolescents’ self-reported symptoms at Grade 6, 77.6% (Grade 7: 81.2%; Grade 9: 81.8%) of the adolescents were characterized as normal, 6.6% (Grade 7: 6.3%; Grade 9: 7.1%) as borderline, and 15.8% (Grade 7: 12.5%; Grade 9: 11.1%) as clinically elevated.

Comparisons between included (*n* = 634 parents, *n* = 634 adolescents) and excluded (*n* = 284 parents, *n* = 707 adolescents) participants showed predominantly significant differences on the variables of interest (Grade 6: unsupportive anger socialization: *t* [904] = 3.49; parent-report internalizing: *t* [907] = 5.74; parent-report externalizing: *t* [907] = 7.03; adolescent-report internalizing: *t* [1301] = 2.52; adolescent-report externalizing *t* [1302] = 4.96; *p*s < 0.01), with the exception of dysfunctional anger regulation (*t* [904] = 1.06, *p* = 0.29). Directions of effects showed that included families reported less unsupportive anger socialization and less internalizing/externalizing problems. Moreover, families with lower HISEI (*t* [929] = 5.44), with children attending the lowest-track school (*χ*^2^ [1,1325] = 126.46, *p* < 0.01), and in which German was spoken less frequently (*t* [1317] = 8.79, showed a higher dropout (*p*s < 0.01).

### Measures

#### **Parents’ Unsupportive Anger Responses (Parent-Report)**

 The Emotions as a Child Scale (EAC, O'Neal & Magai, 2005) was used to assess how parents responded to adolescents’ anger. Parents are asked to rate how often they use specific supportive (i.e., rewarding reactions) and unsupportive strategies (i.e., neglecting, overriding, magnifying, or punishing reactions; e.g., “When my teen is angry, I usually ignore him/her”). Each strategy comprises three items. We used a German translation (Otterpohl et al., 2012), which, compared with the original EAC parent self-report version, was slightly modified in two respects: Firstly, since one item of the subscale *override* (“When my teen is angry, I buy him/her something (s)he likes.”) showed unacceptable factor loadings in other studies (e.g., Guo et al., 2017), we removed this item from the questionnaire. Secondly, as the original EAC comprises only three items on supportive reactions, we added the following extra item to the *reward* scale: “When my teen is angry, I show him/her that I accept his/her anger.”

A further modification was the use of a four (instead of the typically used five) point Likert-type scale (1 = *almost never*, 2 = *sometimes*, 3 = *often* 4 = *almost always*) in order to standardize test administration to be consistent with the range of other measures also administered. From all items, we computed one *Total Unsupportive Anger Socialization Score*, including the 11 items that represent unsupportive reactions and the 4 reversed *reward* items that originally represented supportive reactions. This was done because combining the subscales allowed one overall cross-lagged panel model to be created across three measurement points. Internal consistency for the *Total Unsupportive Anger Socialization Score* was good for all measurement points (α _Grade 6_ = 0.76; α _Grade 7_ = 0.77; α _Grade 9_ = 0.79).

Both the original and the modified version have shown to be internally consistent in samples of children and adolescents with alphas ranging from 0.73 to 0.90. Scores have been correlated with parental stress, parent–child connectedness, child–parent conflicts as well as children’s trait emotion regulation and negative emotions during acute social stress in samples with children and adolescents (Guo et al., 2017; Otterpohl et al., 2012).

#### **Adolescents’ Dysfunctional Anger Regulation (Parent-Report)**

The *Questionnaire for the Measurement of Emotion Regulation in Children and Adolescents* (FEEL-KJ; Grob & Smolenski, 2009) measures adolescent-reported use of several emotion regulation strategies in response to anger, sadness, and fear. In the present study, we rephrased items into the third person (e.g., “When my teen is angry, (s)he tries to change the things which make him/her angry.”) to assess parent-reports on adolescents’ emotion regulation. The original and also the modified version have shown factorial two-dimensionality, internal consistencies ranging from 0.64 to 0.93, and correlations with parenting dimensions as well as depressive and anxiety symptoms in both community and clinical samples (Grob & Smolenski, 2009; Otterpohl et al., 2012). The scale includes 14 items that represent *adaptive (Behavioral Problem-solving*, *Distraction*, *Mood-raising*, *Acceptance*, *Forgetting, Cognitive Problem-solving*, and *Reappraisal)* and 10 items representing *maladaptive (Resign*, *Venting*, *Withdrawal*, *Self-defeat*, and *Rumination)* anger regulation strategies. Parents answered the items on a 4-point Likert-type scale (1 = *almost never*, 4 = *almost always*). In order to obtain a total score, the items for adaptive strategies were reversed and the mean score of all items was calculated (hereinafter referred to as: *Dysfunctional Anger Regulation*). Internal consistency was very good and comparable to the norm sample (α _Grade 6_ = 0.85; α _Grade 7_ = 0.86; α _Grade 9_ = 0.87).

#### **Adolescents’ Internalizing and Externalizing Problems (Parent and Adolescent Report)**

The German version of the *Strengths and Difficulties Questionnaire* (SDQ; Goodman, 1997) is a brief behavioral screening questionnaire for children and adolescents between 3 and 16 years old. It has shown comparability to the English version with regard to its factor structure, reliability (α _Total difficulties_ = 0.82), and validity (ability to distinguish adolescents drawn from community and clinic samples and between adolescents with diagnosed hyperactivity, conduct, and emotional disorders) for a norm sample of 930 students (Klasen et al., 2000). The questionnaire consists of five subscales *Emotional Symptoms* (e.g., “I worry a lot”), *Conduct Problems* (“I take things that are not mine from home, school, or elsewhere”), *Hyperactivity/Inattention* (“I’m easily distracted, I find it difficult to concentrate”), and *Peer Relationship Problems* (“I would rather be alone than with people of my age”) with *five items per subscale, rated from 0* = *not true to 2* = *certainly true*. In the case of nonclinical samples, Goodman, Lamping, and Ploubidis (2010) suggest combining the subscales of emotional problems and peer relationship problems to provide a measure of *Internalizing problems*, and conduct problems and inattention/hyperactivity to provide a measure of *Externalizing Problems.* For the current study, both parent-reports and adolescent-reports were obtained. Internal consistency was good for parent-reported internalizing problems (α _Grade 6_ = 0.75, α _Grade 7_ = 0.76, α _Grade 9_ = 0.73) and externalizing problems (α _Grade 6_ = 0.79, α _Grade 7_ = 0.79, α _Grade 9_ = 0.76) and acceptable to good for adolescent-reported internalizing problems (α _Grade 6_ = 0.72, α _Grade 7_ = 0.68, α _Grade 9_ = 0.68) and externalizing problems (α _Grade 6_ = 0.77, α _Grade 7_ = 0.72, α _Grade 9_ = 0.75).

### Data Analytic Strategy

To determine the concurrent, predictive, and stability links among the variables, we conducted a series of manifest cross-lagged models in *Mplus* Version 8 (Muthén & Muthén, 1998–2017). The basic idea of these models is that initial levels of the dependent variable are controlled, and thus the focus is on predicting change in the dependent construct over and above initial levels (Selig & Little, 2012). In all models, we used the MLR estimator implemented in Mplus 8 which produces parameter estimates with standard errors and a mean-adjusted chi-square test statistic that are robust to non-normality. Missing data were handled by full information maximum likelihood estimation (FIML).

**Fig. 1:**
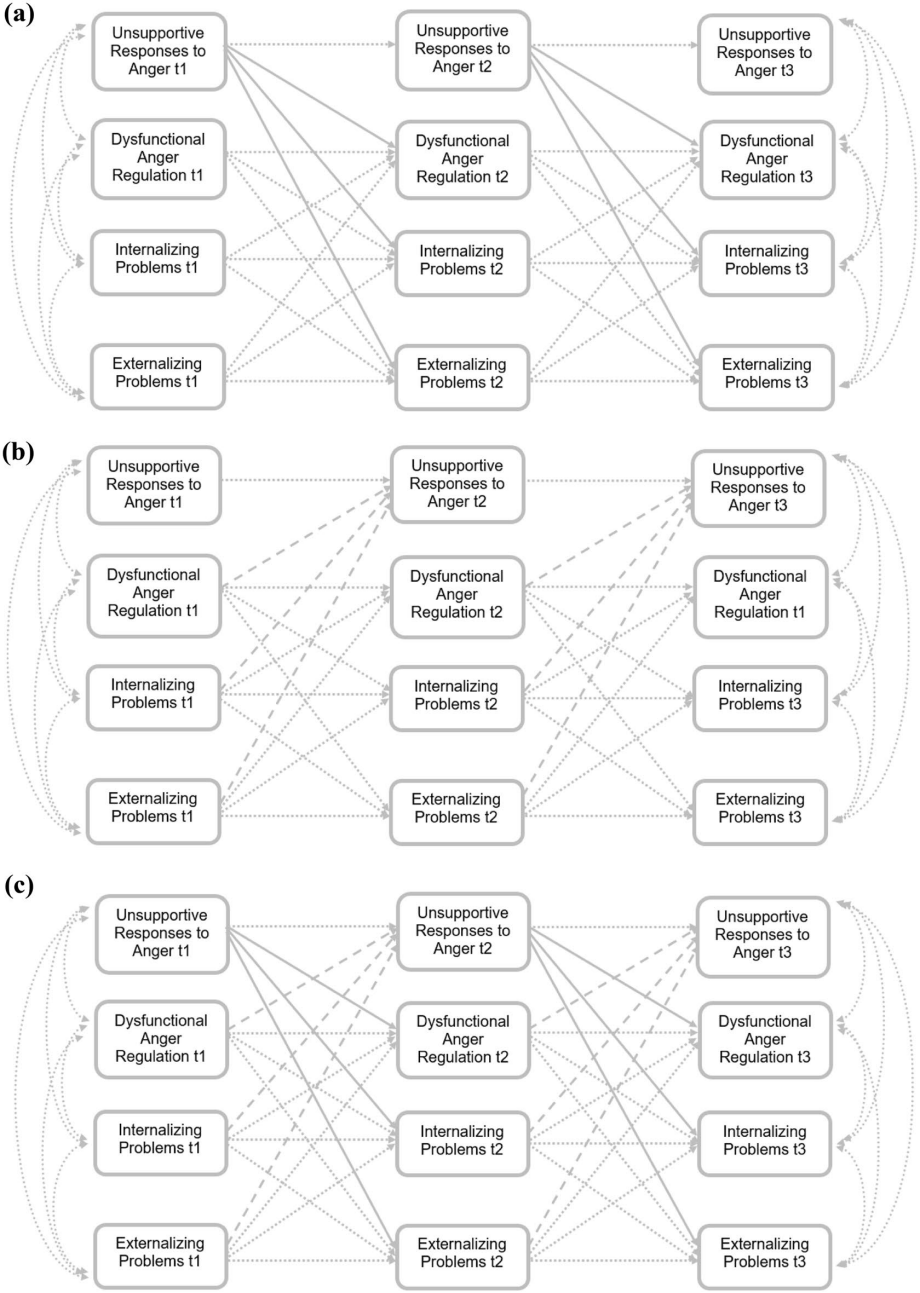
Theoretical Models of **(a)** Parent-directed effects, **(b)** Adolescent-directed effects, and **(c)** Reciprocal effects (Parent-directed + Adolescent-directed). Dotted lines indicate stabilities over time and cross-lagged effects between anger regulation, internalizing, and externalizing problems. Continuous lines indicate parent-directed effects of emotion socialization. Dashed lines indicate adolescent-directed effects

Following Cole and Maxwell’s (2003) suggestions, we tested and compared several manifest cross-lagged models to test our first hypothesis on reciprocal relations among anger socialization, anger regulation, and internalizing/externalizing problems. More precisely, we created three different models (see Fig. 1) which represented different ideas on directionality behind the respective constructs: In the first model, we conducted a *parent-directed model* in which parental unsupportive responses predicted subsequent adolescent dysfunctional anger regulation and internalizing/externalizing problems. Thereafter, we conducted an *adolescent-directed model*, which included adolescent dysfunctional anger regulation and adolescent internalizing/externalizing problems predicting parental unsupportive responses to anger. These models were compared with a *reciprocal model* (the combination of *parent-directed and adolescent-directed* model). As depicted in Fig. 1, all models controlled for stabilities and concurrent relations among all constructs, and also for reciprocal effects of dysfunctional anger regulation and internalizing/externalizing problems from Grade 6 to Grade 7, and Grade 7 to Grade 9, respectively. Thus, the only difference between the three models was the direction of effect of parents’ unsupportive responses to anger.

Importantly, due to statistical constraints (e.g., suppression effects) we did not model a fully cross-lagged model which would have included additional paths from T1 to T3 (i.e., from T1 parents’ responses to anger to T3 internalizing/externalizing problems and vice versa). Since the first and the second model were nested in the *reciprocal model*, it was possible to compare them with the reciprocal model according to several fit indices, such as scaled *χ*^2^ difference test (Satorra & Bentler, 2001), Comparative Fit Index (*CFI),* Root Mean Square Error of Approximation *(RMSEA)*, Standardized Root Mean Squared Residual (*SRMR*; for an overview, see Hu & Bentler, 1999), and Akaike information criterion *(AIC)*. With respect to model fit, Hu and Bentler (1999) recommended values close to 0.95 for *CFI*, 0.06 for *RMSEA,* and 0.08 for *SRMR* as cutoff criteria for a relatively good fit. With regard to the *AIC* criterion, there is no fixed cut-off value indicating a good model fit. Rather, the values of the tested models are compared, with a relatively lower *AIC* value indicating a better model fit.

All analyses were conducted twice to include the multi-informant outcome variables regarding adolescents’ symptoms (parent-reports vs. adolescent self-reports on adolescent internalizing/externalizing problems). All other variables were assessed from the parents’ perspective in both models.

## Results

### Preliminary Analyses

Mean scores, standard deviations, and correlations among variables are presented in Table 2 and Table 3. All variables were relatively stable (0.38 < *r*s < 0.71) and (with few exceptions) significantly intercorrelated in expected directions. Correlations with SES (0.01 ≤ *r*s ≤ 0.15) were small or non-significant. Thus, we did not control for SES. Adolescent gender (-0.03 ≤ *r*s ≤ 0.26) was predominantly related to externalizing behavior, with boys having higher mean scores than girls. Accordingly, we integrated the influence of gender on externalizing and internalizing problems in our models, but this procedure did not show any significant changes. Moreover, we conducted additional multi-group analyses to examine whether relations were different for boys and girls. Prior to testing the structural equation models, all variables were screened for normality and outliers. No variables exceeded the cutoff values of 2 for skewness and 7 for kurtosis (West et al., 1995).

### Final Models

Comparison of the three different theoretical models (Fig. 1) showed a good model fit for both the *parent-directed and adolescent-directed models* (for both parent-report and adolescent-report; *CFI*s > 0.98; *RMSEA*s < 0.07; *SRMR*s < 0.06), but as expected in our original hypothesis, they were significantly worse than the *reciprocal model*, respectively (see Table 4). In line with results on the other fit indices, the *AIC* values were lowest for the reciprocal model (for both parent-report and adolescent-report). Based on these comparisons, we used the reciprocal models to interpret cross-lagged effects in more detail.

#### Model with Parent-Reports on Adolescents’ Externalizing and Internalizing Problems

**Fig. 2:**
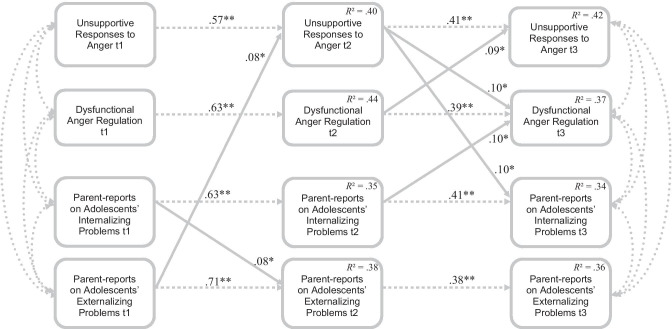
Final path model with Parent-reports on Adolescents’ Externalizing and Internalizing Problems (Bidirectional; *χ*^2^ [*df* = 10, *N* = 611] = 19.32; *p* < 0.05; CFI = 1.00; RMSEA = 0.04; SRMR = 0.01). Dotted lines indicate concurrent relations and stabilities over time. Continuous lines indicate cross-lagged effects. All coefficients are standardized. For the sake of clarity only significant paths are depicted and cross-sectional relations at t2 are not depicted. The model tested includes all paths depicted in Fig. 1c. ** *p* < 0.01, * *p* < 0.05

 First, externalizing problems at Grade 6 predicted unsupportive responses to anger at Grade 7 (*β* = 0.08), controlling for unsupportive responses to anger at Grade 6, which, in turn, predicted subsequent adolescent dysfunctional anger regulation (*β* = 0.10) and internalizing problems (*β* = 0.10) at Grade 9, controlling for prior levels at Grade 7 (indirect effects not significant). Results are depicted in Fig. 2. Second, over and above stabilities, internalizing problems at Grade 6 predicted externalizing problems at Grade 7 (*β* = 0.08), but externalizing problems were not predictive for any of the other variables at Grade 9. Third, dysfunctional anger regulation at Grade 7 predicted unsupportive responses to anger at Grade 9 (*β* = 0.09) over and above stabilities, indicating reciprocal relations between these constructs. Finally, internalizing problems at Grade 7 predicted dysfunctional anger regulation at Grade 9 (*β* = 0.10), controlling for prior levels at Grade 7.

The moderating effect of gender was investigated using multi‐group analysis. Two models were compared using a Satorra-Bentler *χ*2 difference test: In the first model, path coefficients were set equal for girls and boys, while in the second model paths were allowed to vary between the two groups. Multi‐group analyses revealed no significant improvement when including adolescents’ gender as a grouping variable (*χ*2‐diff [66] = 72.79, *p* = 0.26).

#### Model with Adolescent Self-Reports on Externalizing and Internalizing Problems

**Fig. 3:**
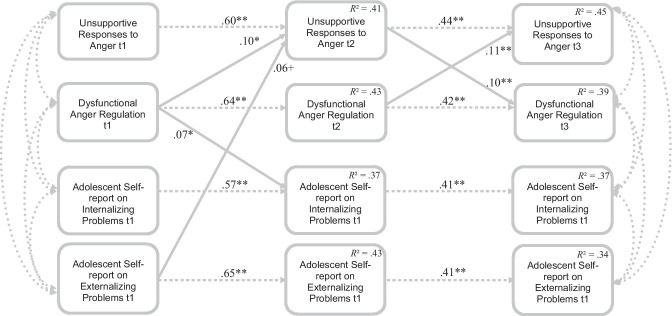
Final path model with Adolescent Self-reports on Externalizing and Internalizing Problems (Bidirectional; *χ*^2^ [*df* = 10, *N* = 634] = 8.06; *p* = 0.62; CFI = 1.00; RMSEA = 0.00; SRMR = 0.01). Dotted lines indicate concurrent relations and stabilities over time. Continuous lines indicate cross-lagged effects. All coefficients are standardized. For the sake of clarity only significant paths are depicted and cross-sectional relations at t2 are not depicted. The model tested includes all paths depicted in Fig. 1c. ** *p* < 0.01, * *p* < 0.05, + *p* < 0.10

 Consistent with the results described above, externalizing problems at Grade 6 predicted unsupportive responses to anger at Grade 7 (*β* = 0.06, *p* < 0.10), which in turn predicted dysfunctional anger regulation (*β* = 0.10) at Grade 9, over and above stabilities (indirect effect not significant). Vice versa, dysfunctional anger regulation at Grade 6 predicted unsupportive responses to anger at Grade 7 (*β* = 0.10), which in turn predicted dysfunctional anger regulation (*β* = 0.10) at Grade 9 (indirect effect not significant). Additionally, dysfunctional anger regulation at Grade 6 predicted internalizing problems at Grade 7 (*β* = 0.07*)*, after prior levels were controlled. Finally, dysfunctional anger regulation at Grade 7 predicted unsupportive responses to anger at Grade 9 (*β* = 0.11), over and above stabilities (Fig. 3). Again, multi‐group analyses revealed no better fit for the model including gender as a grouping variable (*χ*2‐diff [66] = 61.75, *p* = 0.62).

## Discussion

The goals of our study were to examine cross-lagged relations between parental responses to anger, adolescents’ anger regulation, and internalizing/externalizing problems. Different model comparisons supported our original hypothesis that the reciprocal model would be superior to alternate unidirectional models, indicating a close interplay of the respective constructs. However, a different pattern of results emerged with respect to internalizing and externalizing problems, as per our expectations in our second hypothesis.

### Longitudinal Relations Among Parental Responses to Anger, Adolescent Dysfunctional Anger Regulation, and Internalizing/Externalizing Problems

Predictive paths consistently indicated predominantly adolescent-directed effects between *externalizing problems* and unsupportive responses to anger. Moreover, externalizing problems consistently predicted later internalizing problems (directly in the adolescent self-reports on symptoms and indirectly via parents’ anger socialization in the parent-reports on adolescents’ symptoms). With respect to *internalizing problems*, less consistent results were found. For the model with parent-reports on adolescents’ symptoms, a relatively clear picture of parent-directed effects emerged. In contrast, the model with adolescent self-reports on symptoms indicated adolescent-directed effects.

These findings provide interesting insight regarding the interplay between the developmental trajectories of internalizing and externalizing problems from early to mid-adolescence. Although externalizing problems are known to often co-occur with internalizing problems, mechanisms underlying this co-occurrence in adolescence are still unclear. The model with adolescent self-reports on symptoms suggests that dysfunctional anger regulation increases the likelihood of later comorbid internalizing problems but not vice versa. This result resembles findings from a recent study with younger children (Oh et al., 2020). However, it should be noted that results from our model with parent-reports on adolescents’ symptoms showed the opposite picture. These discrepant findings mirror the low consistencies in both parents’ and adolescents’ assessment of adolescent psychological adjustment that have been observed for many years (De Los Reyes & Kazdin, 2005). One of many explanations may be that parents and adolescents build their assessment on different experiences of problems within shared (e.g., at home) and non-shared (e.g., at school, time spent with peers) environments. Our results emphasize once more that multi-informant studies are considered to be the gold standard because each perspective provides unique information. However, in the present study, the adolescent-reports on internalizing problems may be more accurate as adolescents become better at masking emotions and withdraw from interactions with parents, whereas parent-reports on externalizing may be more trustworthy due to social desirability biases in self-reports regarding items on aggression or delinquency.

Regarding the role of *dysfunctional anger regulation*, reciprocal relations between unsupportive responses to anger and dysfunctional anger regulation may be interpreted as a vicious circle in which adolescents’ problems in dealing with anger serve as a maintaining factor for non-optimal parenting and vice versa. As emphasized by Morris et al. (2007), assumptions in the emotion socialization framework have been mainly derived from studies with younger children, due to a lack of studies with older children. Our results are in line with Brenning’s cross-lagged finding that the interplay between parenting, emotion regulation, and adjustment appears to be more complex and that results on the mediating role of emotion regulation are not as clear as expected. Instead, bidirectional relations may play an even greater role during adolescence, suggesting that this assumption (already included in emotion socialization framework; Eisenberg et al., 1998; Morris et al., 2007) should be emphasized much more. More longitudinal research is needed to shed more light on this research question.

Another striking and rather unexpected finding is that very different results occurred for the paths from Grade 6 to Grade 7 as compared to the path from Grade 7 to Grade 9. While parents’ unsupportive responses to anger at the beginning of adolescence had no predictive value for the development of adolescent outcomes over time, parents’ responses to anger and adolescents’ anger regulation were more closely interwoven in the later course of adolescence. Generally, our findings support the assumption that the importance of parents’ responses to anger seems to become increasingly important over the course of adolescence. However, this finding needs to be interpreted with caution, due to the fact that the time span between the waves was significantly different, as no data could be collected in the 8^th^ grade.

### Implications

Our findings have several important implications for clinical child and adolescent psychology. From our results, the positive message to parents and practitioners might be that emotion socialization opportunities for parents are still likely to have an impact on adolescent development, although adolescent-directed effects seem to be gaining importance. Although (or for the reason that) the parent–child relationship during the teenage years is characterized by many emotional challenges, these changes also offer the opportunity to nurture even closer relationships. Examples could be that parents tell their teens about their own experiences when they were the same age or teach them strategies to better tolerate distressing emotions. This can strengthen emotional guidance and also contribute to feelings of closeness and intimacy. Thus, it is key to develop and provide parenting programs that target parental emotion socialization, such as *Tuning in to Teens* (Havighurst et al., 2015) or *Attachment-Based Family Therapy* (Diamond et al., 2014) and/or work with teens to teach them functional anger regulation strategies with programs such as *Dialectical Behavior Therapy for Adolescents* (Miller et al., 2007).

With respect to cross-cultural implications, our study contributes to a better understanding of emotion socialization in different countries. To date, very little is known about emotion socialization during adolescence in German families. In research on general parenting constructs (e.g., parental psychological control), there is some controversy about whether specific parenting strategies can be seen as universally detrimental across nations and cultures (e.g., Pomerantz & Wang, 2009). Some argue that the effects of controlling parenting are moderated by cultural orientation (e.g., Chao & Aque, 2009). Therefore, it is possible that in some cultures that endorse parental unsupportive reactions to anger (e.g., overriding or ignoring reactions that aim to minimize adolescents’ expression of anger), such practices would be more acceptable and less strongly associated with adolescent difficulties. Research with German families found they were less emotionally expressive compared with families in other Western cultures (Matsumoto et al., 2008) and tended to talk less openly about their emotions (Croucher et al., 2015). Thus, it could be argued that unsupportive reactions to anger may be less detrimental in German families. However, and in line with a study by Di Giunta et al. (2020) comparing the development of adolescents in nine different countries (with individualistic and collectivistic cultures), our findings support the assumption that the functionality of different emotion-related parenting practices is generalizable to other individualistic cultures such as the German one.

### Strengths and Limitations

Overall, this study has important strengths. This is one of the first studies to examine longitudinal relations among emotion socialization, emotion regulation, and psychosocial adjustment in adolescence using cross-lagged analyses that make it more likely that significant changes in outcomes can be ascribed to the intended predictors. Another strength is the simultaneous inclusion of internalizing and externalizing problems that allows conclusions to be drawn on the transdiagnostic importance of anger socialization. A final strength is the inclusion of parent and adolescent reports on adolescents’ symptoms.

Despite these strengths, our study has several limitations. First, as is typical for cross-lagged models in which stabilities and concurrent relations are controlled: effect sizes were relatively small and pattern of results were not always straight-forward (in the way that predicting variables at T1 predicted change in a variable at T2, which in turn predicted change in the outcome variables at T3). A second weakness is that due to space limitations in the adolescents’ questionnaires (which already included other constructs for another broader research question), we were not able to assess all constructs from both the parent and the adolescent perspective at all three waves. Moreover, many participants (31%) of the original sample were excluded since longitudinal data was missing, resulting in a culturally less diverse and therefore less representative sample. Particularly, the model may not hold for adolescents attending lowest-track schools, families with lower SES, in which German is less frequently spoken or families with extreme forms of unsupportive anger socialization. Thus, it should be noted that although distribution of adolescents’ internalizing/externalizing indicated representativeness of the included families in this regard (Klasen et al., 2000), our study belongs to the long list of studies in which the majority of subjects were WEIRD (i.e., Western [and White], educated, industrialized, rich, and democratic; Henrich et al., 2010) despite the fact that most humans do not fit this description. Based on increasing proposals to address research-related discrimination against subjects in terms of race, ethnicity, SES, etc. (e.g., Usher, 2018), an important goal of future studies in the field of emotion socialization should be to collect samples with greater diversity to increase the generalizability of findings in this field. A further limitation is that in the present study no concrete clinical disorders were considered, which would allow more specific conclusions on the implications of anger socialization and anger regulation for different diagnoses.

In summary, our study suggests that anger socialization and anger regulation may be transdiagnostic risk factors that play a role across adolescents’ internalizing and externalizing problems, although their interplay may have different underlying processes. In future research, further studies are needed to compare patterns of results among different emotions and to shed light on further moderating factors, in order to draw reliable conclusions on the important questions concerning causal relations and transdiagnostic implications of parents’ emotion socialization during adolescence, which as yet, is still in its infancy.

**Table 1:** Number of overall participants, number of adolescent-parent-dyads included, and dropout of included dyads

Participants		Grade 6	Grade 7	Grade 9
Adolescents	invited participating adolescents adolescent-parent-dyads	1,763 1,341 918		
Adolescent-Parent-Dyads	adolescent-report parent-report	634 611	631 588	631 537
	Inclusion rate	69%		
	Dropout (compared to baseline assessment at Grade 6)		0.48% 3.77%	0.48% 12.12%

**Table 2:** Means and Standard Deviations for Parent and Adolescent Reports

	*M*_parent_	*SD*_parent_	*M*_child_	*SD*_child_
(1) G6 Parent-report on Parental Responses to Anger	1.67	0.34	–	–
(2) G7 Parent-report on Parental Responses to Anger	1.67	0.35	–	–
(3) G6 Parent-report on Parental Responses to Anger	1.61	0.34	–	–
(4) G6 Parent-report on Adolescents’ Dysfunctional Anger Regulation	2.36	0.35	–	–
(5) G7 Parent-report on Adolescents’ Dysfunctional Anger Regulation	2.40	0.36	–	–
(6) G9 Parent-report on Adolescents’ Dysfunctional Anger Regulation	2.32	0.37	–	–
(7) G6 Adolescents’ Internalizing Problems*	1.36	1.41	2.60	1.70
(8) G7 Adolescents’ Internalizing Problems*	1.39	1.45	2.47	1.63
(9) G9 Adolescents’ Internalizing Problems*	1.32	1.41	2.57	1.58
(10) G6 Adolescents’ Externalizing Problems*	1.98	1.55	3.22	1.84
(11) G7 Adolescents’ Externalizing Problems*	1.97	1.61	3.21	1.74
(12) G9 Adolescents’ Externalizing Problems*	1.72	1.44	2.99	1.72

*G6* sixth grade, *G7* seventh grade, *G9* ninth grade

*assessed as parent-report and adolescent-report

**Table 3:** Internal Consistencies and Intercorrelations (Parent Reports on Adolescents’ Problems below and Adolescent Self-reports above the Diagonal)

Adolescent Parent	(1)	(2)	(3)	(4)	(5)	(6)	(7)	(8)	(9)	(10)	(11)	(12)	(13)	(14)	α _Parent_	α _Child_
(1) Adolescent Sex									-0.07	-0.15**	-0.23**	0.18**	0.14**	0.10**	–	–
(2) G5 HISEI	-0.03								0.09*	0.04	0.04	0.10*	0.08*	0.09*	–	–
(3) G6 Unsup Resp	0.09*	0.07							0.10*	0.02	0.07	0.11**	0.12**	0.07	0.76	–
(4) G7 Unsup Resp	0.10*	0.11**	0.63**						0.06	0.03	0.06	0.16**	0.17**	0.13**	0.77	–
(5) G9 Unsup Resp	0.15**	0.10*	0.55**	0.63**					0.07	0.05	0.10*	0.19**	0.16**	0.20**	0.79	–
(6) G6 Dysf Ang Reg	0.09*	0.02	0.23**	0.25**	0.25**				0.18**	0.17**	0.14**	0.23**	0.17**	0.17**	0.85	–
(7) G7 Dysf Ang Reg	0.14*	0.01	0.16**	0.31**	0.30**	0.65**			0.10*	0.12**	0.08	0.20**	0.18**	0.15**	0.86	–
(8) G9 Dysf Ang Reg	0.01	0.04	0.21**	0.28**	0.42**	0.53**	0.59**		0.12*	0.10*	0.15**	0.16**	0.17**	0.22**	0.87	–
(9) G6 Internalizing	0.11**	0.15**	0.27**	0.26**	0.29**	0.36**	0.27**	0.28**	0.39**	0.29**	0.28**	0.12**	0.12**	0.06	0.75	0.72
(10) G7 Internalizing	0.08	0.12**	0.23**	0.34**	0.28**	0.28**	0.33**	0.30**	0.67**	0.38**	0.30**	0.10*	0.13**	0.05	0.76	0.68
(11) G9 Internalizing	0.05	0.15**	0.25**	0.31**	0.38**	0.27**	0.27**	0.41**	0.58**	0.62**	0.35**	0.15**	0.17**	0.13**	0.73	0.68
(12) G6 Externalizing	0.25**	0.13**	0.36**	0.32**	0.36**	0.34**	0.28**	0.26**	0.36**	0.28**	0.30**	0.41**	**0.40**	0.33**	0.79	0.77
(13) G7 Externalizing	0.26**	0.12**	0.26**	0.41**	0.34**	0.31**	0.34**	0.22**	0.34**	0.39**	0.34**	**0.75**	0.43**	0.34**	0.79	0.72
(14) G9 Externalizing	0.21**	0.07	0.22**	0.34**	0.39**	0.19**	0.24**	0.37**	0.27**	0.29**	0.42**	**0.65**	**0.66**	0.46**	0.76	0.75

Coefficients in bold represent stabilities. Coefficients italics represent consistencies (adolescent vs. parent report) on internalizing/externalizing problems at the same time point

*Unsup Resp* Unsupportive Responses to Anger, *Dysf Ang Reg* Dysfunctional Anger Regulation. *G6* sixth grade, *G7* seventh grade, *G9* ninth grade. ^1^Coding: Girls 0, boys 1

*p < 0.05; **p < 0.01

**Table 4:** Comparison of Cross-Lagged Models

	*χ*^*2*^* (df)*	Comparison	SB scaled ^*∆*^*χ*^*2*^* (df)*	*AIC*	*CFI*	*RMSEA*	*SRMR*
Model with Parent-reports on Adolescents’ Externalizing and Internalizing Problems							
(a) Parent-directed model (Fig. 1a)	57.57 (16)	(a) vs. (c)	38.48** (6)	11,993.33	0.98	0.06	0.05
(b) Adolescent-directed model (Fig. 1b)	31.99* (16)	(b) vs. (c)	12.62* (6)	11,967.43	0.99	0.04	0.02
(c) Reciprocal model (Fig. 1c)	19.32** (10)	–	–	11,965.04	1.00	0.04	0.01
Model with Adolescent Self-reports on Externalizing and Internalizing Problems							
(a) Parent-directed model (Fig. 1a)	32.71** (16)	(a) vs. (c)	25.74** (6)	14,881.86	0.99	00.04	0.04
(b) Adolescent-directed model (Fig. 1b)	20.80** (16)	(b) vs. (c)	12.90* (6)	14,869.58	1.00	0.02	0.02
(c) Reciprocal model (Fig. 1c)	8.06** (10)	–	–	14,867.85	1.00	0.00	0.01

*CFI* Confirmatory Fit Index, *RMSEA* Root Mean Square Residual, *SRMR* Standardized Root Mean Squared Residual, *AIC* Akaike Information Criterion

*p < 0.05; **p < 0.01

## References

[CR1] Bariola E, Gullone E, Hughes EK (2011). Child and adolescent emotion regulation: The role of parental emotion regulation and expression. Clinical Child & Family Psychology Review.

[CR2] Bailen NH, Green LM, Thompson RJ (2019). Understanding emotion in adolescents: A review of emotional frequency, intensity, instability and clarity. Emotion Review.

[CR3] Beauchaine TP, Cicchetti D (2019). Emotion dysregulation and emerging psychopathology: A transdiagnostic, transdisciplinary perspective. Development and Psychopathology.

[CR4] Brenning K, Soenens B, Van Petegem S, Vansteenkiste M (2015). Perceived maternal autonomy support and early adolescent emotion regulation: A longitudinal study. Social Development.

[CR5] Buckholdt KE, Parra GR, Jobe-Shields L (2014). Intergenerational transmission of emotion dysregulation through parental invalidation of emotions: Implications for adolescent internalizing and externalizing behaviors. Journal of Child and Family Studies.

[CR6] Bundesministerium für Bildung und Forschung [German Federal Ministry of Education and Research] (Ed.) (2008): Berufsbildungsbericht. Bonn.

[CR7] Campos JJ, Mumme D, Kermoian R, Campos RG (1994). A functionalist perspective on the nature of emotion. Japanese Journal of Research on Emotions.

[CR8] Chao RK, Aque C (2009). Interpretations of parental control by Asian immigrant and European American youth. Journal of Family Psychology.

[CR9] Cole DA, Maxwell SE (2003). Testing meditational models with longitudinal data: Questions and tips in the use of structural equation modeling. Journal of Abnormal Psychology.

[CR10] Cracco E, Goossens L, Braet C (2017). Emotion regulation across childhood and adolescence: Evidence for a maladaptive shift in adolescence. European Child & Adolescent Psychiatry.

[CR11] Croucher, S. M., Sommier, M., Rahmani, D., & Appenrodt, J. (2015). A cross- cultural analysis of communication apprehension: A comparison of three European nations. *Journal of Intercultural Communication*, 38.

[CR12] De Los Reyes A, Kazdin AE (2005). Informant discrepancies in the assessment of childhood psychopathology: A critical review, theoretical framework, and recommendations for further study. Psychological Bulletin.

[CR13] Diamond, G. S., Diamond, G. M., & Levy, S. (2014). *Attachment-based family therapy*. APA.

[CR14] Di Giunta, L., Rothenberg, WA., Lunetti, C., Lansford, JE., Pastorelli, C., Eisenberg, N., Peña Alampay, L. (2020) Longitudinal associations between mothers’ and fathers’ anger/irritability expressiveness, harsh parenting, and adolescents’ socioemotional functioning in nine countries. *Developmental Psychology*, *56,* 458–474. 10.1037/dev0000849 PMC7041852 32077717

[CR15] Dodge KA (2006). Translational science in action: Hostile attributional style and the development of aggressive behavior problems. Development and Psychopathology.

[CR16] Eccles JS, Wigfield A, Irving JL (1997). Young adolescent development. What current research says to the middle level practitioner.

[CR17] Ehrenreich-May J, Chu BC (2013). Overview of transdiagnostic mechanisms and treatments for youth psychopathology.

[CR18] Eisenberg N (2020). Findings, issues, and new directions for research on emotion socialization. Developmental Psychology.

[CR19] Eisenberg N, Cumberland A, Spinrad TL (1998). Parental socialisation of emotion. Psychological Inquiry.

[CR20] Folk JB, Zeman JL, Poon JA, Dallaire DH (2014). A longitudinal examination of emotion regulation: Pathways to anxiety and depressive symptoms in urban minority youth. Child and Adolescent Mental Health.

[CR21] Ganzeboom HBG, De Graaf PM, Treiman DJ (1992). A standard international socio-economic index of occupational status. Social Science Research.

[CR22] Gottman, J. M., & DeClaire, J. (1997). *The heart of parenting: How to raise an emotionally intelligent child*. Simon & Schuster.

[CR23] Gottman JM, Katz LF, Hooven C (1996). Parental meta-emotion philosophy and the emotional life of families: Theoretical models and preliminary data. Journal of Family Psychology.

[CR24] Goodman, A., Lamping, D. L., & Ploubidis, G. B. (2010). When to use broader internalising and externalising subscales instead of the hypothesised five subscales on the Strengths and Difficulties Questionnaire (SDQ): data from British parents, teachers and children. *Journal of Abnormal Child Psychology, 38,* 1179–1191. 10.1007/s10802-010-9434-x 20623175

[CR25] Goodman R (1997). The Strengths and Difficulties Questionnaire: A research note. Journal of Child Psychology and Psychiatry.

[CR26] Gross JJ, Thompson RA, Gross JJ (2007). Emotion regulation: Conceptual foundations. Handbook of Emotion Regulation.

[CR27] Grob A, Smolenski C (2009). *Fragebogen zur Erhebung der ER bei Kindern und Jugendlichen (FEEL-KJ)* [Questionnaire for the measurement of emotion regulation in children and adolescents].

[CR28] Guo J, Mrug S, Knight DC (2017). Factor structure of the Emotions as a Child Scale in late adolescence and emerging adulthood. Psychological assessment.

[CR29] Havighurst SS, Kehoe CE, Harley AE (2015). Tuning in to teens: Improving parental responses to anger and reducing youth externalizing behavior problems. Journal of Adolescence.

[CR30] Henrich J, Heine SJ, Norenzayan A (2010). Beyond WEIRD: Towards a broad-based behavioral science. Behavioral and Brain Sciences.

[CR31] Hoffman ML (2000). Empathy and moral development: Implications for caring and justice.

[CR32] Hu L, Bentler PM (1999). Cutoff criteria for fit indexes in covariance structure analysis: Conventional criteria versus new alternatives. Structural Equation Modeling.

[CR33] Kazdin, A. E. (2003). Problem-solving skills training and parent management training for conduct disorder. In A. E. Kazdin & J. R. Weisz (Eds.), *Evidence-based psychotherapies for children and adolescents* (p. 241–262). The Guilford Press.

[CR34] Kehoe CE, Havighurst SS, Harley AE (2020). Tuning in to Teens: Investigating moderators of program effects and mechanisms of change of an emotion focused group parenting program. Developmental Psychology.

[CR35] Klasen H, Woerner W, Wolke D, Meyer R, Overmeyer S, Kaschnitz W (2000). Comparing the German versions of the Strengths and Difficulties Questionnaire (SDQ-Deu) and the Child Behavior Checklist. European Child and Adolescent Psychiatry.

[CR36] Lemerise EA, Arsenio WF (2000). An integrated model of emotion processes and cognition in social information processing. Child Development.

[CR37] Lengua LJ (2006). Growth in temperament and parenting as predictors of adjustment during children’s transition to adolescence. Developmental Psychology.

[CR38] Linehan MM (1993). Skills training manual for treating borderline personality disorder.

[CR39] Maliken AC, Katz LF (2013). Exploring the impact of parental psychopathology and emotion regulation on evidence-based parenting interventions: A transdiagnostic approach to improving treatment effectiveness. Clinical Child and Family Psychology Review.

[CR40] Matsumoto D, Yoo SH, Nakagawa S (2008). Culture, emotion regulation, and adjustment. Journal of Personality and Social Psychology.

[CR41] Miller, A. L., Rathus, J. H., DuBose, A. P., Dexter-Mazza, E. T., & Goldklang, A. R. (2007). Dialectical behavior therapy for adolescents. *Dialectical behavior therapy in clinical practice: Applications across disorders and settings*, 245–263.

[CR42] Morris AS, Silk JS, Steinberg L, Myers S, Robinson LR (2007). The role of the family context in the development of emotion regulation. Social Development.

[CR43] Muthén, L. K., & Muthén, B. O. (1998–2017). *Mplus User*’*s Guide (Eighth Ed.).* Los Angeles, CA: Muthén & Muthén.

[CR44] Oh, Y., Greenberg, M. T., Willoughby, M. T., & Family Life Project Key Investigators (2020). Examining longitudinal associations between externalizing and internalizing behavior problems at within-and between-child levels. Journal of Abnormal Child Psychology.

[CR45] O’Neal & Magai (2005). Do parents respond in different ways when children feel different emotions? The emotional context of parenting. Development and Psychopathology.

[CR46] Otterpohl N, Imort S, Lohaus A, Heinrichs N (2012). Kindliche Regulation von Wut: Effekte familiärer Kontextfaktoren [Anger regulation in children: Effects of contextual factors]. Kindheit und Entwicklung.

[CR47] Otterpohl N, Wild E (2015). Cross-lagged relations among parenting, children's emotion regulation, and psychosocial adjustment in early adolescence. Journal of Clinical Child & Adolescent Psychology.

[CR48] Patterson, G. R. (2016). Coercion theory: The study of change. The Oxford handbook of coercive relationship dynamics, 7–22.

[CR49] Perry NB, Dollar JM, Calkins SD, Keane SP, Shanahan L (2020). Maternal socialization of child emotion and adolescent adjustment: Indirect effects through emotion regulation. Developmental Psychology.

[CR50] Pomerantz EM, Wang Q (2009). The role of parental control in children's development in Western and East Asian countries. Current Directions in Psychological Science.

[CR51] Satorra A, Bentler PM (2001). A scaled difference chi-square test statistic for moment structures analysis. Psychometrika.

[CR52] Selig JP, Little TD, Laursen B, Little TD, Card NA (2012). Autoregressive and cross-lagged panel analysis for longitudinal data. Handbook of developmental research methods.

[CR53] Shenk CE, Fruzzetti AE (2011). The impact of validating and invalidating responses on emotional reactivity. Journal of Social and Clinical Psychology.

[CR54] Steinberg L (2000). The family at adolescence: Transition and transformation. Journal of Adolescent Health.

[CR55] Steinberg L (2001). We know some things: Parent–adolescent relations in retrospect and prospect. Journal of Research on Adolescence.

[CR56] Usher EL (2018). Acknowledging the whiteness of motivation research: Seeking cultural relevance. Educational Psychologist.

[CR57] Valiente, C., Eisenberg, N., Spinrad, T. L., Reiser, M., Cumberland, A., Losoya, S. H., Liew, J. (2006). Relations among mothers’ expressivity, children’s effortful control, and their problem behaviors: a four-year longitudinal study. *Emotion, 6,* 459–472. 10.1037/1528-3542.6.3.459 PMC1676339 16938087

[CR58] West SG, Finch JF, Curran PJ, Hoyle RH (1995). Structural equation models with nonnormal variables: Problems and remedies. Structural equation modeling: Concepts, issues and applications.

[CR59] Werner, K., & Gross, J. J. (2010). Emotion regulation and psychopathology: A conceptual framework. In A. M. Kring & D. M. Sloan (Eds.), *Emotion regulation and psychopathology: A transdiagnostic approach to etiology and treatment* (pp. 13–37). The Guilford Press.

